# Pretreatment Computed Tomography-Based Machine Learning Models to Predict Outcomes in Hepatocellular Carcinoma Patients who Received Combined Treatment of Trans-Arterial Chemoembolization and Tyrosine Kinase Inhibitor

**DOI:** 10.3389/fbioe.2022.872044

**Published:** 2022-05-23

**Authors:** Qianqian Ren, Peng Zhu, Changde Li, Meijun Yan, Song Liu, Chuansheng Zheng, Xiangwen Xia

**Affiliations:** ^1^ Department of Radiology, Union Hospital, Tongji Medical College, Huazhong University of Science and Technology, Wuhan, China; ^2^ Hubei Province Key Laboratory of Molecular Imaging, Wuhan, China; ^3^ Department of Hepatobiliary Surgery, Wuhan No.1 Hospital, Wuhan, China

**Keywords:** radiomics, deep learning, feature robustness, trans-arterial chemoembolization, tyrosine kinase inhibitor, hepatocellular carcinoma

## Abstract

**Aim:** Trans-arterial chemoembolization (TACE) in combination with tyrosine kinase inhibitor (TKI) has been evidenced to improve outcomes in a portion of patients with hepatocellular carcinoma (HCC). Developing biomarkers to identify patients who might benefit from the combined treatment is needed. This study aims to investigate the efficacy of radiomics/deep learning features-based models in predicting short-term disease control and overall survival (OS) in HCC patients who received the combined treatment.

**Materials and Methods:** A total of 103 HCC patients who received the combined treatment from Sep. 2015 to Dec. 2019 were enrolled in the study. We exacted radiomics features and deep learning features of six pre-trained convolutional neural networks (CNNs) from pretreatment computed tomography (CT) images. The robustness of features was evaluated, and those with excellent stability were used to construct predictive models by combining each of the seven feature exactors, 13 feature selection methods and 12 classifiers. The models were evaluated for predicting short-term disease by using the area under the receiver operating characteristics curve (AUC) and relative standard deviation (RSD). The optimal models were further analyzed for predictive performance on overall survival.

**Results:** A total of the 1,092 models (156 with radiomics features and 936 with deep learning features) were constructed. Radiomics_GINI_Nearest Neighbors (RGNN) and Resnet50_MIM_Nearest Neighbors (RMNN) were identified as optimal models, with the AUC of 0.87 and 0.94, accuracy of 0.89 and 0.92, sensitivity of 0.88 and 0.97, specificity of 0.90 and 0.90, precision of 0.87 and 0.83, F1 score of 0.89 and 0.92, and RSD of 1.30 and 0.26, respectively. Kaplan-Meier survival analysis showed that RGNN and RMNN were associated with better OS (*p* = 0.006 for RGNN and *p* = 0.033 for RMNN).

**Conclusion:** Pretreatment CT-based radiomics/deep learning models could non-invasively and efficiently predict outcomes in HCC patients who received combined therapy of TACE and TKI.

## Introduction

In recent years, many novel therapies have modified the therapeutic landscape of hepatocellular carcinoma (HCC) (A. Rizzo et al., 2021; S. De Lorenzo et al., 2018). Furthermore, predictive biomarkers to guide treatment choice were explored extensively (A. Rizzo and G. Rizzo and Brandi, 2021). In particular, trans-arterial chemoembolization (TACE) combined with tyrosine kinase inhibitor molecular targeted therapy has been shown to significantly improve outcomes over TACE alone in patients with HCC (M. Kudo et al., 2020; Z. Peng et al., 2019). Due to tumor heterogeneity, patients’ responses to the combined treatment may vary, indicating exploration of predictors to identify patients who might benefit from the combined treatment is urgently needed (M. Kudo et al., 2020; T. Meyer et al., 2017). Microvascular invasion (MVI) has been proven effective in predicting response to TACE combined with Sorafenib in patients with recurrent intermediate stage HCC (Z. Peng et al., 2019). However, MVI is detected at the resection. Furthermore, tissue-based biomarkers can only reflect the local but not the general characteristics of the heterogeneous nature of the tumor since they mostly rely on a single tumor sample from an approachable lesion in practice. In addition, it is difficult to identify the patient’s current status from an archival sample due to the evolution of the tumor and the tumor microenvironment during anti-cancer treatment. The biomarker to identify patients most likely to benefit from this combined treatment is limited.

Radiomics has been used to evaluate the severity of chronic liver disease and assess the prognosis of malignant liver tumors (S. Chen et al., 2019; G. W. Ji et al., 2019; S. Kim et al., 2019; F. Liu et al., 2018; H. J. Park et al., 2019; X. Xu et al., 2019). Deep learning (DL) has been widely applied to liver imaging for various tasks, including organ segmentation, staging liver fibrosis, tumor detection or classification, and improving image quality (C. A. Hamm et al., 2019; F. Liu F et al., 2019; D. Tamada et al., 2020; K. Wang et al., 2019a and X. Liu Z et al., 2019; K. Wang et al., 2019b and A. Mamidipalli et al., 2019; K. Yasaka et al., 2018a, H. Akai, and O. Abe et al., 2018; K. Yasaka et al., 2018b, H. Akai, and A. Kunimatsu et al., 2018). Because training a DL model with a small sample size for one specific clinical question often does not yield satisfactory results, a machine learning framework that combines radiomics features and deep learning features from pre-trained networks with conventional machine learning methods has satisfying predictive performance accuracy and computational costs for some tasks (S. Raghu et al., 2020).

However, the clinical interpretability and reproducibility of clinical-decision support algorithms remain challenging. The robustness of a radiomics/deep-learning-based prediction model refers to its ability to tolerate perturbation to the image input. Recent studies in natural image processing have revealed that the output of DL models can be easily affected by small-scale perturbations added to the input (P. Malhotra et al., 2021; X. Yuan et al., 2019). Correspondingly, many factors are known to induce variability in radiomics features, including noise (D. Mackin et al., 2018), heterogeneous voxel size (M. Shafiq-Ul-Hassan et al., 2018), variability in imaging protocols, different vendors, image reconstruction processes (M. Meyer et al., 2019), Region of Interest (ROI) segmentation (I. Fotina et al., 2012; C. Haarburger et al., 2020; J. Kalpathy-Cramer et al., 2016; Q. Qiu et al., 2019), patient motion, overall image quality as well as tumor phenotype (J. E. van Timmeren et al., 2016).

To the best of our knowledge, this is the first work performing a high-throughput benchmark analysis, along with a feature robustness analysis, to predict short-term tumor response and overall survival in patients with HCC who treated with TACE in combination with targeted molecular therapy.

## Materials and Methods

### Data/Population and Data Acquisition

The ethics committee of our institute approved the study and waived written informed consent due to the retrospective design.

We reviewed the electronic medical records of HCC patients who received combined treatment of TACE and TKI from Sep. 2015 to Dec. 2019 at our institute (Union Hospital, Tongji Medical College, Huazhong University of Science and Technology). The inclusion criteria were as follows: 1) age, ≥ 20 years; 2) tumors confined to the liver without macro-vascular invasion or extra-hepatic metastasis; 3) tumors are measurable by the modified Response Evaluation Criteria in Solid Tumours (mRECIST); 4) Eastern Cooperative Oncology Group (ECOG) performance status of 0 or 1, Child-Pugh scores ≤7 points and adequate organ function. Those without complete medical records or high-quality CT images in electronic format were excluded.

Two radiologists reviewed pre-treatment and post-treatment CT images to evaluate short-term tumor response according to the mRECIST. Any inconsistency of assessment results was resolved by consensus. Tumor response was evaluated every 8 weeks. Overall survival (OS) was defined as the time from the date of treatment to the date of death without regarding the cause of death, and censored at the date of last follow-up for survivors. The regimen of TACE plus TKI, response evaluation, clinical data and CT data acquisition are detailed in Supplementary Material.

### Tumor Segmentation and Imaging Pre-Processing

The Region of interest (ROI) of primary tumor, defined as enhanced area in arterial phase CT images in accordance with mRECIST, was manually delineated by two experienced radiologists (XW X and QQ R) using a 3D Slicer software (A. Fedorov et al., 2012). To be consistent with deep learning features, three consecutive slices with the maximum cross-sectional area of the tumor lesion were selected. The two observers repeated the same procedures 2 weeks later and any disagreement was resolved through consultation. The brightness, the size and of the image were standardized and the noise in the image was removed using the methods reported in literature (H. Koyuncu and Ceylan, 2018). In brief, resegmentation refers to the process whereby only pixels within a specified grey value range (−1,000, 400) are retained to exclude irrelevant organs and objects. The CT images’ appropriate window wide and center were adaptively adjusted based on the tumor region’s Hounsfield unit values. The images were then subjected to imaging normalization (the intensity of the image was scaled to 0–255) to avoid data heterogeneity bias. Histogram equalization was used to improve the brightness and contrast of the image for practitioners to analyze. CT images are mainly affected by quantum noise, arising from the variability of the electronic density of tissue voxels, statistically represented by a random Gaussian process. We used Gaussian filter to remove the noise in the image. The images with informative slices (three consecutive axial slices with maximum tumor area) corresponding to the segmented tumor region were cropped to 224 mm × 224 mm using a bounding box spanning the whole tumor area.

### Feature Extraction

Six commonly used pre-trained convolutional neural networks (CNNs) (Y. Hu et al., 2021; T. N. Sainath et al., 2015), including InceptionResNetV2, InceptionV3, Resnet50, VGG16, VGG19, and Xception, were pretrained on ImageNet, which contains a large number of object categories and manually annotated training images. When performing deep learning feature extraction, we treated the pre-trained network as an arbitrary feature extractor, allowing the input image to propagate forward, stopping at the pre-specified layer, and taking the outputs of that layer as our features. After removing the last fully connected layer, we got feature maps of CT images with the maximum area of the tumor lesion, which corresponded to location invariance in the input layer. After global pooling, each feature map vector was transformed to a maximal raw value. The representational deep learning features refer to a total of 2048 (Resnet50, InceptionV3, and Xception), 1,536 (InceptionResNetV2) or 512 (VGG16, VGG19) features were converted from feature maps to numeric values.

Handcrafted radiomics features were automatically computed from the radiologist-drawn ROIs using the Pyradiomics package implemented in Python. Defined radiomics features with or without wavelet filtration were extracted in accordance with feature definitions described by the image biomarker standardization initiative (IBSI) reporting guidelines (A. Zwanenburg et al., 2020). Features were divided into three groups: (I) first-order statistics; (II) shape features; and (III) second-order features: gray level co-occurrence matrix (GLCM), gray level run length matrix (GLRLM), gray level size zone matrix (GLSZM), gray level dependence matrix (GLDM), neighborhood gray tone difference matrix (NGTDM).

### Feature Robustness Evaluation

The ROI images were adjusted to evaluate the impact of perturbations on feature robustness. We tested three perturbations as follows: 1) slice thickness (S): CT images were reconstructed contiguously at 1, 2, 3 and 5 mm section thicknesses; 2) rotation (R): The image and mask were rotated in the axial (x, y) plane, over a set angle θ [−30°, −15°, 15°, and 30°]; 3) segmentation (Seg): ROIs were automatically expanded or shrinked by 20% (A. Zwanenburg et al., 2019).

The Intra-class Correlation Coefficient ICC was chosen to ensure absolute agreement and not only consistency across perturbations. According to the guidelines (T. K. Koo and Li, 2016), all the features with an ICC of more than 0.85 for all tested perturbations were selected for further study analysis. Raw feature vectors were further standardized by centering on the mean and scaling to unit variance.

### Feature Selection of Informative Features and Predictive Model Construction

To further reduce feature dimension, the following steps were performed: 1) removing robust features with zero median absolute deviation (MAD); 2) only considering the top 20% features selected by univariate analysis; 3) algorithm-based feature selection; 4) the wrapper feature selection method based on the recursive feature addition algorithm to select the most predictive features. The features were fed to machine learning classifiers and the performance was evaluated by the area under the receiver operating characteristic curve (AUC). A 10-fold cross validation was used in the feature dimension step to avoid data leakage and overestimation.

The algorithm-based feature selectors included ReliefF (RELF), Fischer Score (FSCR), Gini index (GINI), Chisquare score (CHSQ), joint mutual information (JMI), conditional infomax feature extraction (CIFE), double input symmetric relevance (DISR), mutual information maximization (MIM), conditional mutual information maximization (CMIM), interaction capping (ICAP), *t*-test score (TSCR, only for binary classification), minimum redundancy maximum relevance (MRMR), and mutual information feature selection (MIFS). These selectors take a filter-method approach for feature selection. The filter method filters out the irrelevant feature and redundant columns from the model by using different metrics through ranking.

Twelve supervised machine learning classifiers, including Nearest Neighbors, Support Vector Classifiers (SVC) with linear or radial basis function (RBF) kernels, Gaussian processes, decision trees, random forests, multilayer perceptrons, AdaBoost, naïve Bayes, quadratic discriminant analysis (QDA), XGBoost, and logistic regression, were then used to train models for predicting short-term disease control. These classifiers were all imported from scikit-learn implemented in Python (version 3.6.4) (A. Abraham et al., 2014). During the model debugging, samples were shuffled to ensure data randomization. We adopted the Synthetic minority over-sampling technique (SMOTE) (N. V. Chawla et al., 2002), one of the commonly-used oversampling algorithms, to achieve class balance during the cross-validation step.

The terminology of each predictive model was consistent with its feature exactor, selector, and classifier. For example, VGG19_FSCR_QDA was a model trained by the QDA classifier, with features selected by FSCR and exacted by VGG19. The predictive performance of the models and their stability was evaluated by the AUC and relative standard deviation (RSD), respectively. RSD was calculated according to the formula: RSD = (sd_AUC_/mean_AUC_) × 100, where sd_AUC_ and mean_AUC_ were the standard deviation and mean of the ten cross-validated AUC values, respectively. Accuracy, sensitivity, specificity, precision, and F1 score were also calculated to further evaluate the selected model (Sokolova and Japkowicz, 2006).

### Statistical Analysis

Continuous variables with normal distribution were presented as mean ± SD (standard deviation) and those with abnormal distribution were presented as median (range). The continuous variables were compared using the *t* test or Kruskal-Wallis tests. Non-continuous variables were compared using the Pearson X^2^ test or Fisher’s exact test.

Survival curves were plotted using the Kaplan-Meier method and compared using the log-rank test. Cox proportional hazard analysis was used to identify factors associated with survival. A *p*-value of less than 0.05 was considered statistically significant.

## Results

### Patient Demographics

A total of 103 HCC patients (92 males and 11 females; age (mean ± SD): 52 ± 9 years) who received combined treatment of TACE and TKI were enrolled in this study. Of these, 72 were identified as disease control (1complete tumor response, 54 partial tumor response, and 17 stable diseases) based on mRECIST, yielding a disease control rate (DCR) of 69.9%. The rest were identified as progressed disease (PD, 30.1%). Clinical and tumor characteristics for all patients are listed in Table 1. The clinical and tumor characteristics differences between PD and non-PD groups are statistically insignificant.

**TABLE 1:** Baseline demographic and clinical characteristics of patients.

Characteristic	Total (*n* = 103)	PD (*n* = 31)	Non PD (*n* = 72)	*p* Value
Age (year), (mean ± SD)	52 ± 9	52 ± 8	52 ± 10	0.732
Sex				0.730
Male, n (%)	92 (89.3%)	27 (87.1%)	65 (90.3%)	
Female, n (%)	11 (10.7%)	4 (12.9%)	7 (9.7%)	
ECOG score				0.375
0, n (%)	88 (85.4%)	25 (80.6%)	63 (87.5%)	
1, n (%)	15 (14.6%)	6 (19.4%)	9 (12.5%)	
Aetiology				0.978
Hepatitis B, n (%)	83 (80.6%)	25 (80.6%)	58 (80.6%)	
Hepatitis C, n (%)	14 (13.6%)	4 (12.9%)	10 (13.9%)	
Nonviral hepatitis, n (%)	6 (5.8%)	2 (6.5%)	4 (5.6%)	
Child-Pugh classification				1.000
Child-Pugh A, n (%)	92 (89.3%)	28 (90.3%)	64 (88.9%)	
Child-Pugh B ≤ 7, n (%)	11 (10.7%)	3 (9.7%)	8 (11.1%)	
BCLC stage				0.720
B, n (%)	94 (91.3%)	29 (93.5%)	65 (90.3%)	
C, n (%)	9 (8.7%)	2 (6.5%)	7 (9.7%)	
Maximum tumor diameter(mm), median (range)	59.68 (10.40–153.33)	70.26 (10.40–153.33)	56.10 (13.34–144.21)	0.081
AFP				0.983
≤400 ng/ml, n (%)	53 (51.5%)	16 (51.6%)	37 (51.4%)	
>400 ng/ml, n (%)	50 (48.5%)	15 (48.4%)	35 (48.6%)	

**FIGURE 1:**
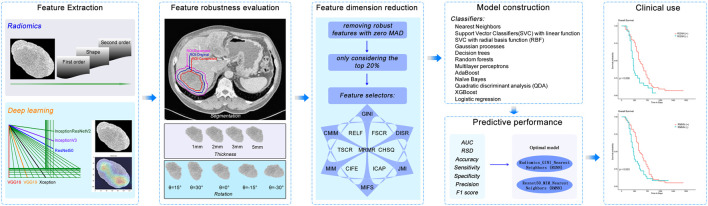
Workflow of major steps in the current work. Tumors are segmented manually and pre-processed. Features are extracted with handcrafted radiomics and six popularly used pre-trained deep learning CNNs, respectively. ICC meters the robustness of features for each perturbation type (segmentation, thickness, and rotation). Robust features are then used to construct models for predicting short-term disease control of tumors by combining each of 13 feature selectors and 12 machine learning classifiers. The best-performing model is evaluated for predicting overall survival.

Our study setup consists of three parts: 1) feature extraction and robustness analysis; 2) constructing models for predicting disease control, performance analysis, and identification of optimal models; 3) OS prediction performance analysis using the optimal models. Figure 1 shows the workflow of the study.

### Feature Robustness Evaluation

A consistency test was applied to evaluate feature robustness. Imaging perturbations produced a slight impact on the stability of radiomics features, with ICC of 0.93 ± 0.11 for S, 0.94 ± 0.15 for R, and 0.96 ± 0.22 for Seg, respectively. High stability was also observed in S and R perturbations for deep learning features extracted with Rnest50, with ICC of 0.89 ± 0.09 and 0.86 ± 0.12, respectively. However, Seg perturbations had moderate impact on the stability of deep learning features extracted from Rnest50, with an ICC of 0.80 ± 0.14. The results of robustness evaluation for features from all extractors were summarized in Supplementary Table S1.

**FIGURE 2:**
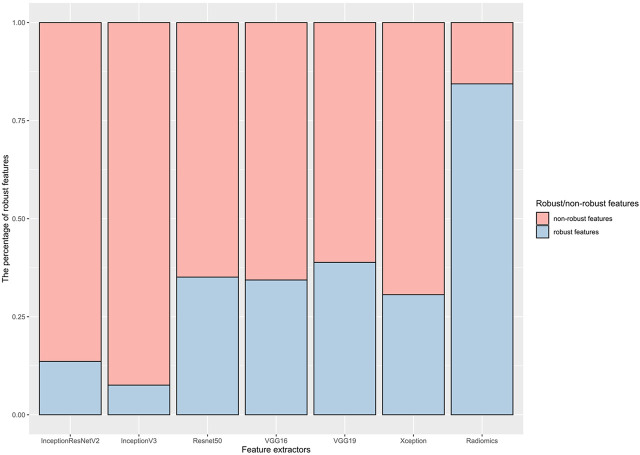
The percentage of robust features against image perturbation.

Figure 2 shows the results of feature robustness analysis with ICC cutoff value of 0.85. There were 718/851 (84.37%) robust features in radiomics group. In deep learning group, the highest percentage robust features is 38.87% (199/512) from VGG19 by using the same cutoff value of ICC, followed by 35.11% (719/2048) from Resnet50, 34.38% (176/512) from VGG16, 30.62% (627/2048) from Xception, 13.61% (209/1,536) from InceptionResNetV2, and 7.57% (155/2048) from InceptionV3. The results of features robustness analysis with other ICCs were presented in Supplementary Figure S1. These results indicated that radiomics features were more stable than deep learning features; in addition, segmentation perturbation (Seg) seemed produce greater impact on stability in deep learning features.

### Predictive Performance of Radiomics/Deep Learning Models on Short-Term Disease Control

A total of 156 radiomics features-based models and 936 deep learning features-based models were constructed, and those classified by the k Nearest Neighbors have excellent performance for predicting short-term disease control, reached a median value of AUC of 0.85 (range: 0.64–0.94) (Supplementary Figure S2) and median RSD of 1.87 (range: 0.26–11.31).

**FIGURE 3:**
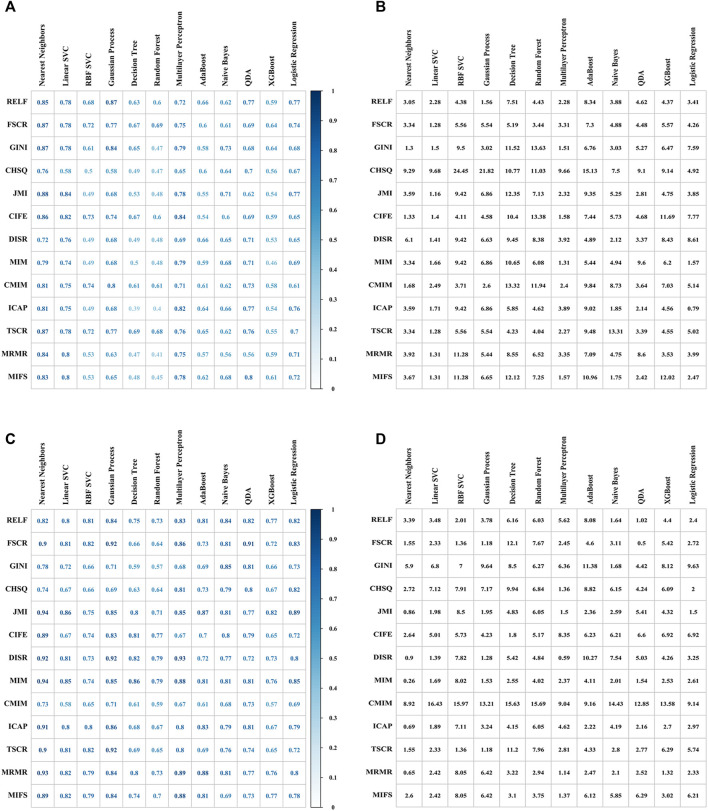
Performance of different combinations of feature selectors (rows) and ML classifiers (columns) for predicting short-term disease control. 10-fold cross-validated AUC values **(A)** and RSD values **(B)** of 156 models with Radiomics features. 10-fold cross-validated AUC values **(C)** and RSD values **(D)** of 156 models with deep learning features extracted from Resnet50.

The Radiomics_GINI_Nearest Neighbors (RGNN) was identified as optimal model in radiomics group, with a cross-validated AUC of 0.87, RSD 1.30, accuracy 0.89, sensitivity 0.88, specificity 0.90, precision 0.87, and F1 score 0.89. (Figures 3A,B). Radiomics_JMI_Nearest Neighbors had a better AUC value of 0.88, but a higher RSD value of 3.59. The Resnet50_MIM_Nearest Neighbors (RMNN) was identified as the optimal model in deep learning group, with a cross-validated AUC of 0.94, RSD 0.26, accuracy 0.92, sensitivity 0.97, specificity 0.90, precision 0.83, and F1score 0.92 (Figures 3C,D). The Resnet50_JMI_Nearest Neighbors had a comparable AUC value 0.94, but a higher RSD value of 0.86.

The list of all feature selectors was in Supplementary Table S2; the ML methods’ parameter settings and tuning range were presented in Supplementary Material. The predictive performance of models constructed by other combinations of CNNs, selectors, and classifiers was Supplementary Figure S3.

### Predictive Performance of Radiomics_GINI_Nearest Neighbors and Resnet50_MIM_Nearest Neighbors on Overall Survival

**FIGURE 4:**
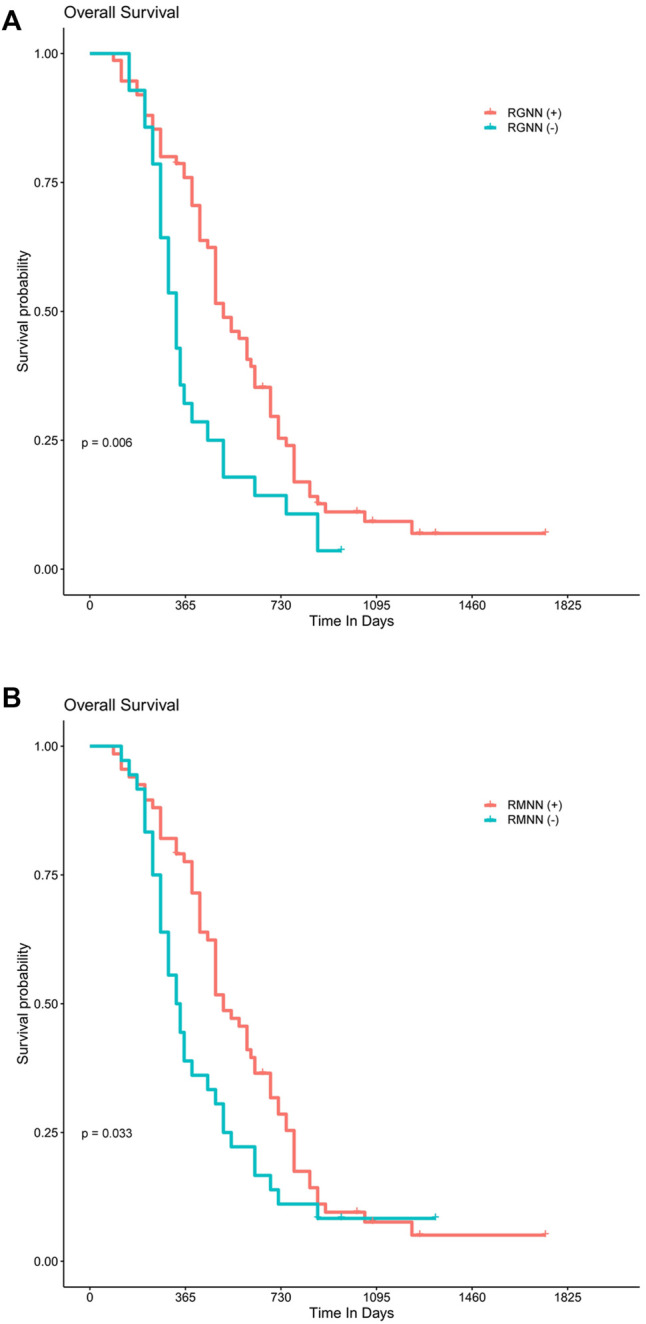
Best-performing model predicting overall survival. Kaplan–Meier survival analysis shows a statistically significant survival advantage for the Radiomics_GINI_Nearest Neighbors **(A)** and Resnet50_MIM_ Nearest Neighbors **(B)**, respectively.

For 99 patients with survival data, the median follow-up time was 15 months (range: 10–24 months). The results of the Kaplan-Meier survival analysis are presented in Figures 4A,B. There was a statistically significant survival advantage for Radiomics_GINI_Nearest Neighbors (*p* = 0.006) and Resnet50_MIM_ Nearest Neighbors (*p* = 0.033). Cox proportional hazard analysis showed that Radiomics_GINI_Nearest Neighbors (HR, 2.49; 95% CI, 1.36–4.55; *p* = 0.003) and Resnet50_MIM_Nearest Neighbors (HR, 1.83; 95% CI, 1.05–3.17; *p* = 0.032) was independently associated with overall survival (Supplementary Table S3).

## Discussion

This study constructed stable radiomics/deep learning models based on a high-throughput analysis for predicting outcomes in HCC patients who received combined treatment of TACE and TKI. We evaluated the robustness of radiomics/deep learning features against multiple perturbations and further evaluated 1,092 combinations of varied feature extractors, selectors, and machine learning techniques. Radiomics_GINI_Nearest Neighbors and Resnet50_MIM_ Nearest Neighbors were identified as the optimal models to predict short-term tumor response and overall survival in two groups (radiomics and deep learning), respectively. Since CT imaging is non-invasive and time-saving, this technique provided us with a fast and auxiliary approach to predict outcomes, thus helping to initially screen patients who might benefit from the combined treatment.

The main idea of deep learning is to employ a deep neural network (DNN) model. To effectively construct deep learning models, we need much more data for training to identify optimal models than prevalent statistical machine learning models. The success of transfer learning schemas, which is frequently used to overcome the limitation of small data sets is clearly contributing to approach DL models as powerful extractors of useful feature sets (H. C. Shin et al., 2016).

Feature robustness depends on the tumor phenotype and is not generalizable (J. E. van Timmeren et al., 2016). In this study, we evaluated the robustness of radiomics and deep learning features by addressing three types of common perturbations, including slice thickness (S), rotation (R), and ROI segmentation (Seg). Our results indicated that Radiomics features seemed more stable than deep learning features in general. To the best of our knowledge, this was the first work to assess the impact of these perturbations on feature stability. The stability of radiomics/deep learning features was more susceptible to Seg. So it is always better performing a “safe” contouring when segmenting, that is, underestimating rather than overestimating the ROI (M. Mottola et al., 2021). These processes can minimize possible variations between centers, machines, image reconstruction methods, and delineation uncertainties. Conducting from these features, our models can be widely applied for CT data obtained in various institutions.

We further investigated 1,092 combinations of feature exactors, feature selectors, and machine learning techniques to construct predictive models. The DL-based model’s prediction ability seemed better than the radiomics-based model. The radiomics-based model with features selected by GINI and classified with Nearest Neighbors was identified to be the optimal model that could effectively predict patients’ outcomes. For deep learning-based models, the combination of Resnet50, MIN, and Nearest Neighbors exhibited high predictive power. These results may be helpful for guidance in choosing a better combination of methods. However, which model is better and more practical still needs further studies to verify.

There are several limitations. First, this study was conducted in a single tertiary hospital; limitations inherent to a retrospective design, including small sample size and selection bias, may have influenced the findings. Furthermore, because of the retrospective character of this work, we used perturbation methods rather than test-retest imaging to evaluate feature robustness. In the future, a prospective test-retest study should be conducted. Secondly, there was no external validation cohort to verify the efficacy of our predictive models. Thirdly, three consecutive sections of the tumor were sampled for analysis, and no volume assessment was performed. In a previous study, it was found that data from a single slice was sufficient for this type of analysis (F. Ng et al., 2013). Apart from that, we only investigated some of the influencing factors affecting the image features. Other factors, such as image reconstruction methods, noise removal methods, and histogram equalization approaches, need further studies. Fifthly, although our results demonstrated strong prediction performance, implying that transfer learning might address domain differences, there was heterogeneity across the source and destination databases. Deep learning models explicitly developed for HCC were required. Additionally, the findings’ interpretability is a ubiquitous limitation when developing any artificial intelligence or machine learning model applied to medical imaging (F. Cabitza et al., 2017; Z. Liu Z et al., 2019; R. Sun et al., 2018). The issue of findings’ interpretability should be improved and solved in further studies.

We believe that the proposed radiomic/deep learning based machine learning model is applicable to other modalities, outcomes, and diseases, with certain modality-specific perturbations. Further research involving standardization across various scanner parameters could aid in harmonizing image attributes in advance. Another major obstacle in this research area is the development of an extensive public database with sufficient annotated medical imaging data to train plenty of parameters in the neural network. Such a database will dramatically help provide more clinically relevant features to train models with better performance.

## Conclusion

This study constructed stable predictive models from radiomics/deep learning features based on pre-treatment CT imaging using high-throughput analysis. These models could effectively predict short-term tumor response and overall survival in HCC patients who received combined treatment of TACE and targeted molecular therapy. Since CT imaging is non-invasive and time-saving, this technique provided us with a fast and auxiliary approach to identify patients who might benefit from the combined treatment and have the potential to improve precision oncology.

## Data Availability

All data generated or analyzed during this study are included in this article and its online supplementary files. Further inquiries can be directed to the corresponding author.

## Glossary

AUC: area under the curve
CHSQ: Chisquare score
CIFE: conditional infomax feature extraction
CMIM: conditional mutual information maximization
DISR: double input symmetric relevance
DL: deep learning
FSCR: Fischer Score
GINI: Gini index
GLCM: gray level co-occurrence matrix
GLDM: gray level dependence matrix
GLRLM: gray level run length matrix
GLSZM: gray level size zone matrix
HCC: hepatocellular carcinoma
IBSI: image biomarker standardization initiative
ICAP: interaction capping
JMI: joint mutual information
MAD: median absolute deviation
MIFS: mutual information feature selection
MIM: mutual information maximization
mRECIST: modified Response Evaluation Criteria in Solid Tumours
MRMR: minimum redundancy maximum relevance
NGTDM: neighborhood gray tone difference matrix
QDA: quadratic discriminant analysis
RBF: radial basis function
RELF: ReliefF
RGNN: Radiomics_GINI_Nearest Neighbors
RMNN: Resnet50_MIM_Nearest Neighbors
ROI: Regions of interest
RSD: relative standard deviation in percentile
SVC: support Vector Classifiers
TACE: Trans-arterial chemoembolization
TKI: tyrosinekinase inhibitor
TSCR: *t*-test score
