# Supplementary figures and images for: Pretreatment Computed Tomography-Based Machine Learning Models to Predict Outcomes in Hepatocellular Carcinoma Patients who Received Combined Treatment of Trans-Arterial Chemoembolization and Tyrosine Kinase Inhibitor
Source: Front Bioeng Biotechnol. 2022 May 23;10:872044. doi: 10.3389/fbioe.2022.872044 (PMC9168370; doi:10.3389/fbioe.2022.872044)

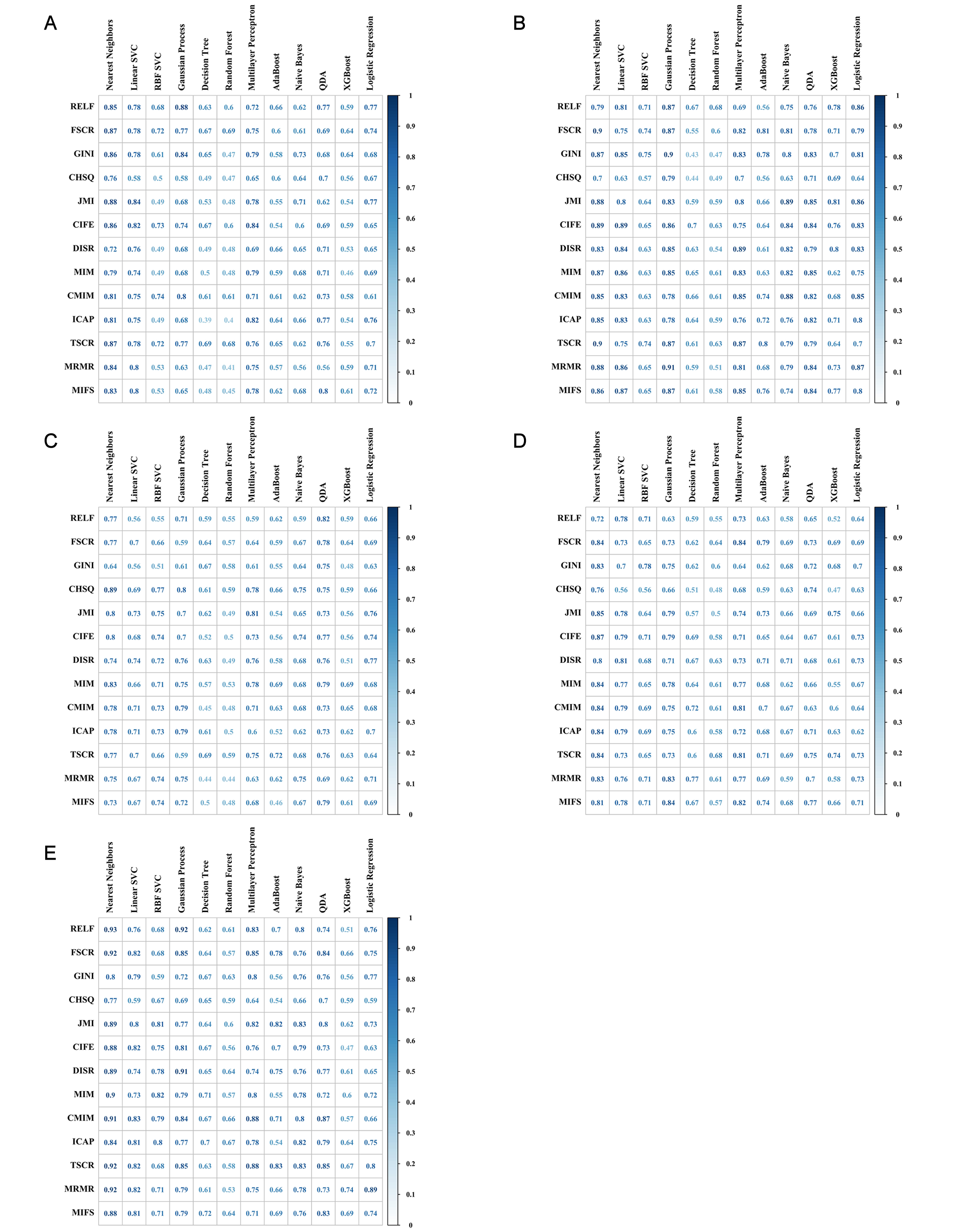

Supplement: Supplementary file 1 [file Image3.jpeg]

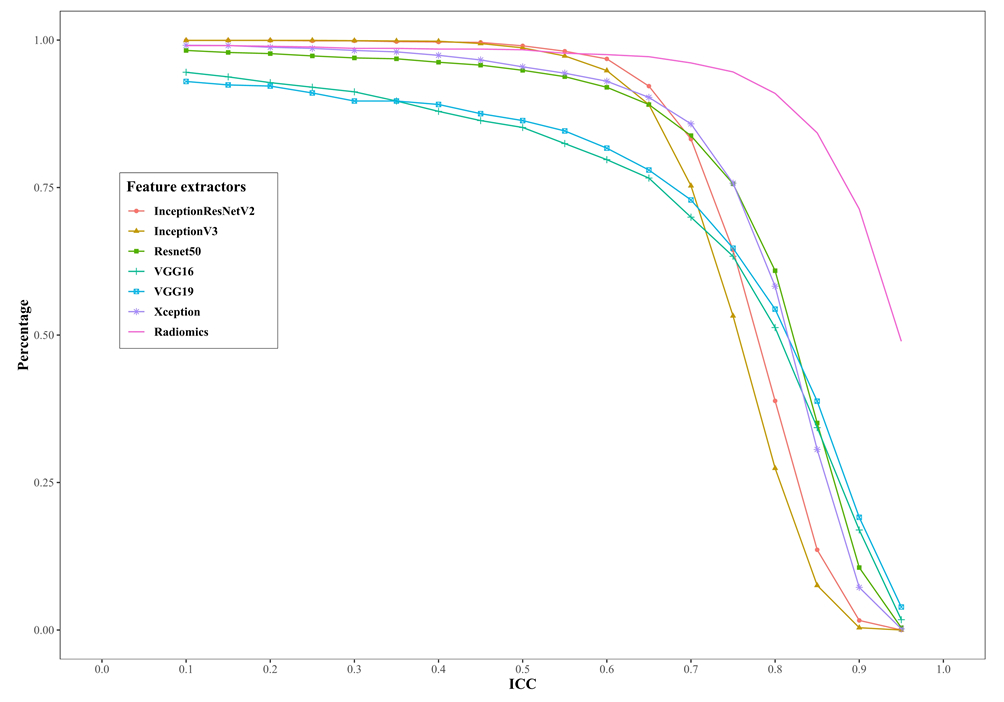

Supplement: Supplementary file 2 [file Image1.jpeg]

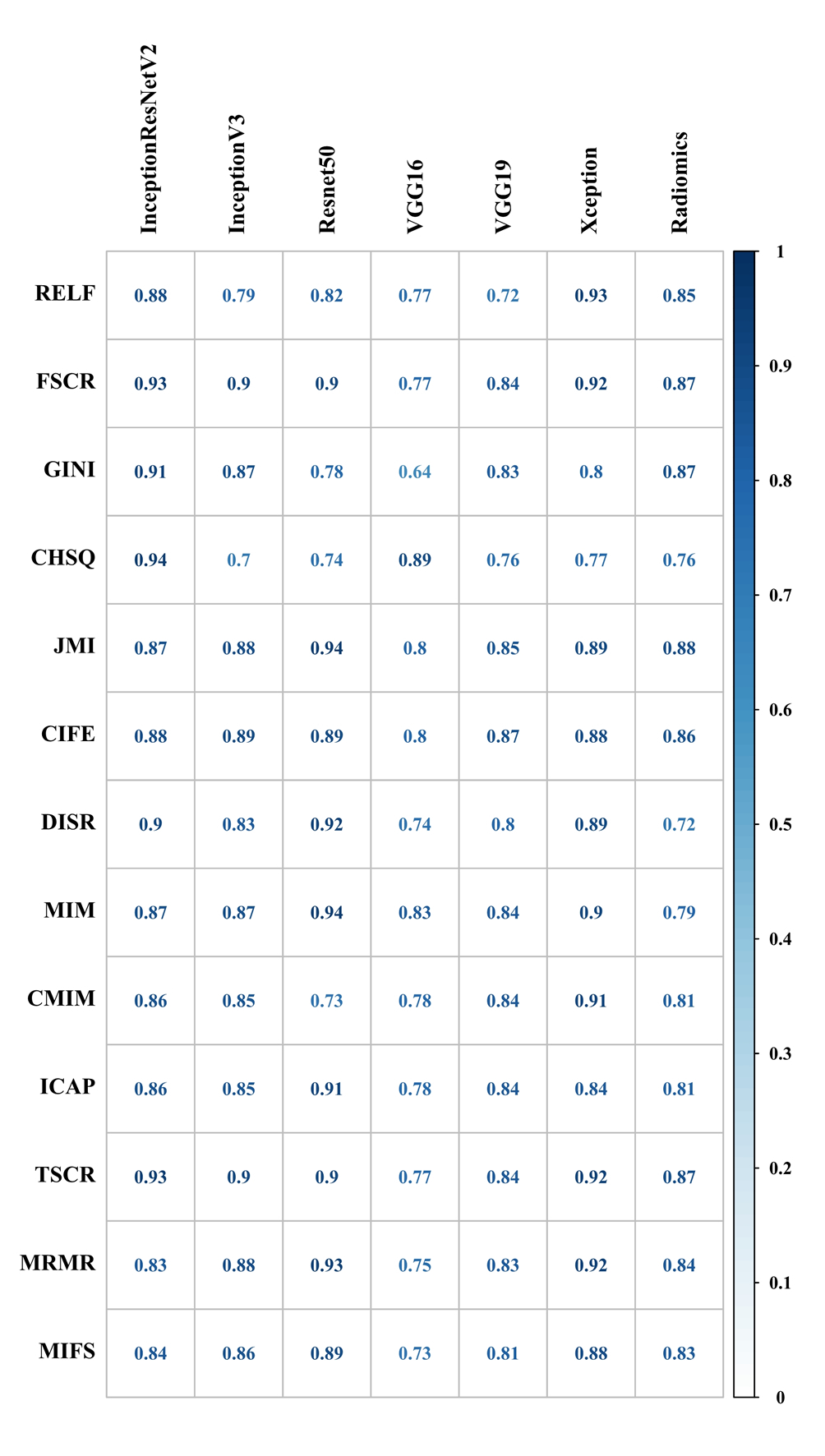

Supplement: Supplementary file 3 [file Image2.jpeg]
