# Supplementary material for: Pretreatment Computed Tomography-Based Machine Learning Models to Predict Outcomes in Hepatocellular Carcinoma Patients who Received Combined Treatment of Trans-Arterial Chemoembolization and Tyrosine Kinase Inhibitor
Source: Front Bioeng Biotechnol. 2022 May 23;10:872044. doi: 10.3389/fbioe.2022.872044 (PMC9168370; doi:10.3389/fbioe.2022.872044)
Supplement: Supplementary file 4 [file DataSheet2.docx]

Supplementary Material

# Supplementary Data

**Tyrosine kinase inhibitors**

TKI (Sorafenib 800 mg/day, Apatinib 500 mg/day, or Lenvatinib 8 mg/day) was initiated 2-3 weeks or 1-3 days prior to the first TACE session, and was discontinued for 2 days before and 2 days after each TACE session. TKI was continued during the entire TACE treatment course until evidence of unacceptable toxicities or untreatable disease progression. Dose reductions and drug interruptions were based on the treatment-related toxicities. Repeated TACE was performed as deemed clinically necessary if the follow-up imaging (ie, dynamic contrast-enhanced CT or MRI 4 weeks after each administration of TACE) showed intrahepatic viable tumor tissue and liver function was adequate to allow for another TACE. If new lesions were found, the patient underwent subsequent TACE. Repeat TACE with the same anticancer agent was recommended when the two-dimensional measurement of the viable lesion was >50% that of the baseline tumour. TACE was repeated for intrahepatic new lesions measuring >10 mm, which show arterial enhancement with venous washout. If venous washout is not associated with arterial enhancement even in lesions >10 mm, TACE was waited until the time venous washout is confirmed.

**Chemoembolization (TACE)**

Hepatic artery infusion chemotherapy was performed by using 300 mg of carboplatin (Bristol-Myers-Squibb, New York, NY). Subsequently, chemolipiodolization was performed by using 50 mg of epirubicin (Pharmorubicin; Pfizer, Wuxi, China) and 8 mg of mitomycin C (Zhejiang Hisun Pharmaceutical, Taizhou, China) mixed with 5 mL of lipiodol (Lipiodol Ultra-Fluide; André Guerbet Laboratories, Aulnay-Sous-Bois, France). Embolization was finally performed with absorbable gelatin sponge particles 1-2 mm in diameter (Gelfoam, Hanzhou Alc, China) until the blood flow was static for more than 10 successive heartbeats. After embolization, angiography was performed to determine the extent of vascular occlusion and to assess blood flow in other arterial vessels. TACE consisted of intra-arterial injection of lipiodol plus epirubicin or miriplatin, followed by injection of an embolic agent (Gelpart) to interrupt blood flow. Selection of anticancer agent (epirubicin or miriplatin) was decided by the sites/investigators; however, the same agent must be used at the repeated TACE sessions. When necessary in treating very large tumours, split TACE was allowed within 4–6 weeks of the first TACE session. First image was taken 4 weeks after split TACE was performed.

**CT data acquisition**

CT data were acquired according to standardized scanning protocols. CT scans uses multi-slice CT system (Philips Healthcare, General Electric Health Care, and Siemens Healthcare) with a tube voltage of 100 to 120 kVp, slice thickness (spacing) of 1 to 5mm, and in-plane resolution of 0.75 x 0.75 mm. CT images were acquired with patients at inspiration breath-hold after contrast injection. We retrieved CT Digital Imaging and Communications in Medicine data from the picture archiving and communication system.

**Clinical data**

Clinical and laboratory data were collected from digital medical records of all patients. Clinical data included age, gender, Barcelona Clinic Liver Cancer (BCLC) stage, Eastern Cooperative Oncology Group (ECOG) performance, Child-Pugh grade. Laboratory data included alpha-fetoprotein (AFP), alanine aminotransferase (ALT), albumin (ALB), total bilirubin (TBIL), prothrombin time (PT), platelet count, absolute neutrophils count (ANC), and absolute lymphocyte count (ALC). Survival data was collected by reviewing records or by following-up.

**Response assessment**

The target lesions observed on two consecutive CT scans were evaluated by the modified Response Evaluation Criteria in Solid Tumours (mRECIST). Patients with progressive disease after treatment (PD) were classified as “PD” and patients with radiographic response, including complete response (CR), partial response (PR) or stable disease were classified as “non PD”.

**Machine learning classifiers**

List of the parameter settings and tuning range for the ML classifiers.

*Nearest Neighbors*: Number of neighbors varied in the range from three to 21, in steps of 3

*Support vector classifier (SVC), with linear kernel*: penalty term C = {0.25, 0.5, 1, 2, 4}

*Support vector classifier, with radial basis function (RBF) kernel*: penalty term C = {0.25, 0.5, 1, 2, 4}, and gamma = {'scale', 'auto', 0.01, 0.1, 1, 10, 100}

*Gaussian process classifier*: kernel set to 1.0 * RBF (1.0)

*Decision tree*: Maximum depth tuned in the range {5, 10, 15, 20}

*Random forests*: 100 trees and maximum number of features set to 'auto'

*Multilayer perceptron*: Maximum iterations set to 5000, alpha = {0.0001, 0.001, 0.01, 0.1, 1, 10}

*AdaBoost*: Default settings

*Naïve Bayes*: Gaussian naïve Bayes classifier, without priors, var_smoothing at default value (1e-9)

*Quadratic discriminant analysis (QDA)*: No priors, covariate estimate regularization at default 0.

*XGBoost*: No instance weighting

*Logistic regression*: default settings (penalty=’l2’, dual=False, tol=0.0001, C=1.0, fit_intercept=True, intercept_scaling=1, class_weight=None, random_state=None, solver=’warn’, max_iter=100, multi_class=’warn’, verbose=0, warm_start=False, l1_ratio=None)

**Image perturbation**

This note provides additional information with regards to the implementation of the image perturbation algorithms. The algorithms were implemented in Python 3.8.5 (Python Software Foundation, Beaverton, Oregon, USA, https://www.python.org/). The implementation drew on functionality offered by the following libraries:

NumPy, referred to as numpy.

SciPy, referred to as scipy.

SimpleITK, referred to as sitk.

**Noise**

scipy. ndimage. gaussian_filter () was used for Gaussian filter.

**Slice thickness**

The spatial resolution in the longitudinal direction is controlled by slice thickness, which influences the tradeoffs among resolution, noise, and radiation dose. The images were resampled using various nominal slice thicknesses from 1 to 5 mm. Resampling was performed by sitk. ResampleImageFilter () and linear interpolation methods were considered.

**Rotation**

Image rotations are used to simulate changes in patient location. The image is rotated in-plane by an angle around the z-axis. The scipy. ndimage. rotate function, which implements rotation as an affine transformation, was used to rotate the image. The intensities in the rotated image are determined using bi-linear sampling. In order to correspond to the expected integer Hounsfield units in CT, intensities are rounded to the nearest integer value after rotation.

The ROI mask rotates in lockstep with the image. The mask's partial volume fractions threshold, on the other hand, is only applied after the image processing scheme's interpolation step.

**Segmentation variation**

Shrinking or growing the segmentation mask is a method to mimic variance in expert delineations. Segmentation variation was performed by scipy. ndimage. binary_dilation () and scipy. ndimage. binary_erosion ().

# Supplementary Figures and Tables

## Supplementary Figures

**Supplementary FIGURE S1.** The percentage of robust features for different ICC thresholds.

**Supplementary FIGURE S2.** Ten fold cross-validated AUCs of 91 models with k Nearest Neighbors classifier.

**Supplementary FIGURE S3.** Ten fold cross-validated AUC values of models with features extracted from other deep learning extractors (A, InceptionResNetV2; B, InceptionV3; C, VGG16; D, VGG19; E, Xception).

## Supplementary Tables

**Table S1 Feature robustness evaluation of perturbations for all extractors**

| **Feature exactor** | **Slice thickness** | **Rotation** | **Variation of ROI** |
| --- | --- | --- | --- |
| InceptionResNetV2 | 0.88±0.04 | 0.90±0.04 | 0.77±0.08 |
| InceptionV3 | 0.87±0.04 | 0.86±0.05 | 0.75±0.08 |
| Resnet50 | 0.89±0.09 | 0.86±0.12 | 0.80±0.14 |
| VGG16 | 0.89±0.12 | 0.79±0.20 | 0.79±0.21 |
| VGG19 | 0.88±0.16 | 0.80±0.21 | 0.80±0.21 |
| Xception | 0.89±0.08 | 0.88±0.09 | 0.79±0.13 |
| Radiomics | 0.93±0.11 | 0.94±0.15 | 0.96±0.22 |

**Table S2 List of all feature selectors and machine learning classifiers**

| **Feature selectors** | **Machine learning classifiers** |
| --- | --- |
| ReliefF (RELF) | Nearest Neighbors |
| Fischer Score (FSCR) | Support Vector Classifiers(SVC) with linear function |
| Gini index (GINI) | SVC with radial basis function (RBF) |
| Chisquare score (CHSQ) | Gaussian processes |
| Joint mutual information (JMI) | Decision trees |
| Conditional infomax feature extraction (CIFE) | Random forests |
| Double input symmetric relevance (DISR) | Multilayer perceptrons |
| Mutual information maximization (MIM) | AdaBoost |
| Conditional mutual information maximization (CMIM) | Naïve Bayes |
| Interaction capping (ICAP) | Quadratic discriminant analysis (QDA) |
| t-test score (TSCR) | XGBoost |
| Minimum redundancy maximum relevance (MRMR) | Logistic regression |
| Mutual information feature selection (MIFS) |  |

**Table S3 Univariate and multivariate survival analysis of overall survival**

|  | Univariate analysis | | | Multivariate analysis | | |
| --- | --- | --- | --- | --- | --- | --- |
| **Factor** | **HR** | **95% CI for HR** | **P value** | **HR** | **95% CI for HR** | **P value** |
| Age | 1.00 | 0.98-1.01 | 0.634 |  |  |  |
| Sex | 1.10 | 0.57-2.14 | 0.772 |  |  |  |
| ECOG score | 1.22 | 0.69-2.17 | 0.494 |  |  |  |
| Aetiology | 0.78 | 0.55-1.10 | 0.158 |  |  |  |
| Child-Pugh classificatin | 1.30 | 0.67-2.52 | 0.429 |  |  |  |
| BCLC stage | 1.24 | 0.62-2.47 | 0.550 |  |  |  |
| AFP (>400ug/ml vs <= 400ug/ml) | 1.25 | 0.83-1.88 | 0.286 |  |  |  |
| Maximum tumor diameter | 1.01 | 1.00-1.01 | 0.003 | 1.01 | 1.00-1.01 | 0.04 |
| Radiomics_GINI_Nearest Neighbors | 2.95 | 1.84-4.73 | 0.000 | 2.49 | 1.36-4.55 | 0.003 |
| Resnet50_MIM_Nearest Neighbors | 2.61 | 1.69-4.02 | 0.000 | 1.83 | 1.05-3.17 | 0.032 |
