# Supplementary material for: Pretreatment Computed Tomography-Based Machine Learning Models to Predict Outcomes in Hepatocellular Carcinoma Patients who Received Combined Treatment of Trans-Arterial Chemoembolization and Tyrosine Kinase Inhibitor
Source: Front Bioeng Biotechnol. 2022 May 23;10:872044. doi: 10.3389/fbioe.2022.872044 (PMC9168370; doi:10.3389/fbioe.2022.872044)
Supplement: Supplementary file 5 [file DataSheet1.docx]

**Table S1 Feature robustness evaluation of perturbations for all extractors**

| **Feature exactor** | **Slice thickness** | **Rotation** | **Variation of ROI** |
| --- | --- | --- | --- |
| InceptionResNetV2 | 0.88±0.04 | 0.90±0.04 | 0.77±0.08 |
| InceptionV3 | 0.87±0.04 | 0.86±0.05 | 0.75±0.08 |
| Resnet50 | 0.89±0.09 | 0.86±0.12 | 0.80±0.14 |
| VGG16 | 0.89±0.12 | 0.79±0.20 | 0.79±0.21 |
| VGG19 | 0.88±0.16 | 0.80±0.21 | 0.80±0.21 |
| Xception | 0.89±0.08 | 0.88±0.09 | 0.79±0.13 |
| Radiomics | 0.93±0.11 | 0.94±0.15 | 0.96±0.22 |

**Table S2 List of all feature selectors and machine learning classifiers**

| **Feature selectors** | **Machine learning classifiers** |
| --- | --- |
| ReliefF (RELF) | Nearest Neighbors |
| Fischer Score (FSCR) | Support Vector Classifiers(SVC) with linear function |
| Gini index (GINI) | SVC with radial basis function (RBF) |
| Chisquare score (CHSQ) | Gaussian processes |
| Joint mutual information (JMI) | Decision trees |
| Conditional infomax feature extraction (CIFE) | Random forests |
| Double input symmetric relevance (DISR) | Multilayer perceptrons |
| Mutual information maximization (MIM) | AdaBoost |
| Conditional mutual information maximization (CMIM) | Naïve Bayes |
| Interaction capping (ICAP) | Quadratic discriminant analysis (QDA) |
| t-test score (TSCR) | XGBoost |
| Minimum redundancy maximum relevance (MRMR) | Logistic regression |
| Mutual information feature selection (MIFS) |  |

**Table S3 Univariate and multivariate survival analysis of overall survival**

|  | Univariate analysis | | | Multivariate analysis | | |
| --- | --- | --- | --- | --- | --- | --- |
| **Factor** | **HR** | **95% CI for HR** | **P value** | **HR** | **95% CI for HR** | **P value** |
| Age | 1.00 | 0.98-1.01 | 0.634 |  |  |  |
| Sex | 1.10 | 0.57-2.14 | 0.772 |  |  |  |
| ECOG score | 1.22 | 0.69-2.17 | 0.494 |  |  |  |
| Aetiology | 0.78 | 0.55-1.10 | 0.158 |  |  |  |
| Child-Pugh classificatin | 1.30 | 0.67-2.52 | 0.429 |  |  |  |
| BCLC stage | 1.24 | 0.62-2.47 | 0.550 |  |  |  |
| AFP (>400ug/ml vs <= 400ug/ml) | 1.25 | 0.83-1.88 | 0.286 |  |  |  |
| Maximum tumor diameter | 1.01 | 1.00-1.01 | 0.003 | 1.01 | 1.00-1.01 | 0.04 |
| Radiomics_GINI_Nearest Neighbors | 2.95 | 1.84-4.73 | 0.000 | 2.49 | 1.36-4.55 | 0.003 |
| Resnet50_MIM_Nearest Neighbors | 2.61 | 1.69-4.02 | 0.000 | 1.83 | 1.05-3.17 | 0.032 |
